# Supplementary material for: Photonics of Trimethine Cyanine Dyes as Probes for Biomolecules
Source: Molecules. 2022 Sep 27;27(19):6367. doi: 10.3390/molecules27196367 (PMC9573451; doi:10.3390/molecules27196367)
Supplement: Supplementary file 1 [file molecules-27-06367-s001.zip › molecules-1912764-supplementary.pdf]

Supplementary Materials
# of the review of P.G. Pronkin and A.S. Tatikolov "Photonics of Trimethine Cyanine Dyes as Probes for Biomolecules"

N.M. Emanuel Institute of Biochemical Physics, Russian Academy of Sciences, Kosygin St. 4, Moscow, 119334 Russia

Corresponding authors: Pavel G. Pronkin pronkinp@gmail.com

Alexander.S. Tatikolov tatikolov@mail.ru

Table S1. Spectral-fluorescent properties of trimethine cyanine dyes in a complex with DNA (in the presence of high concentrations of DNA): maxima of the absorption and fluorescence spectra λ_{abs}(DNA) and λ_{fl}(DNA), respectively, spectral shifts upon the interaction with DNA $\Delta\lambda_{abs}$ and $\Delta\lambda_{fl}$, ratios of fluorescence intensities/quantum yields in the presence and in the absence of DNA I_{fl}(DNA)/I_{fl0} or Φ_{fl}(DNA)/Φ_{fl0}; as well as dye–DNA binding constants K_b and limits of detection/quantification of DNA LOD/LOQ using the dyes as probes

Dye	λ_{abs}(DNA) (~$\Delta\lambda_{abs}$)	λ_{fl}(DNA) (~$\Delta\lambda_{fl}$)	I_{fl}(DNA)/I_{fl0} Φ_{fl}(DNA)/Φ_{fl0}	K_b	LOD/LOQ	Ref.
	nm			L mol^{-1}	μmol L^{-1}	
**1**	541 (6)	563 (-1)	177.6	4.8 × 10^5	–	[24, 27, 40]
**2**	554 (3)	577 (8)	4.59	9.5 × 10^4	–	[27, 32, 33]
**4**	551 (-2)	651 (-6)	2.59	–	–	[27]
**5**	558 (10)	644 (1)	45.0	–	–	
**6**	551 (50)	632 (-4)	2.36	–	–	
**7**	561 (34)	587 (9)	70.9	–	–	
**8**	558 (0)	596 (-119)	71.0	–	–	
**9**	501 (5)	604 (-25)	5.87	–	–	
**10**	506 (6)	583 (-72)	207.4	–	–	
**11**	505 (2)	583 (-89)	222.0	–	–	
**12**	557 (30)	627 (-5)	127.7	–	–	
**13**	515 (-13)1	637 (8)	5.28	–	–	
**15**	575 (11)	582 (8)	23.0	~10^6 ~5 × 10^4	–	[29]
**18**	566 (5)	586 (1)	8.21	1.2 × 10^5	~0.2	[30, 31]
**19**	554 (10)	567 (3)	263.0	1.24 × 10^5	~0.022	
**20**	565 (6)	581 (5)	10	1.2 × 10^5	~1	
**21**	568(8)	580 (6)	6.67	3.7 × 10^4	~2.7	

Dye	λ_{abs}(DNA) (~$\Delta\lambda_{abs}$)	λ_{fl}(DNA) (~$\Delta\lambda_{fl}$)	I_{fl}(DNA)/I_{fl0} Φ_{fl}(DNA)/Φ_{fl0}	K_b	LOD/LOQ	Ref.
	nm			L mol^{-1}	μmol L^{-1}	
**22**	695 (23)	575 (23)	3	–	–	[34]
**23**	581 (-)	618 (-)	150	–	–	[35]
**24**	610 (-)	651 (-)	140	–	–	
**26**	538 (-1)	565 (-5)	15	–	–	[38–41]
**27**	501 (6)	525 (1)	9	–	–	
**28**	550(5)	665 (2)	87	–	–	
**29**	499 (13)	513 (8)	55	–	–	
**30**	504 (11)	518 (7)	41	–	–	
**31**	515 (12)	522 (10)	2.6	–	–	
**38**	–	514 (10)	27.7	–	–	[45]
**39**	–	603 (6)	13.4	–	–	
**40**	–	565 (-3)	132.1	–	–	
**41**	–	573 (4)	60.7	–	–	
**42**	–	449 (-131)	46.8	–	–	
**43**	–	701 (45)	~0.9	–	–	
**44**	–	637 (-35)	76	–	–	
**45**	–	615 (-27)	5.6	–	–	
**46**	–	628 (-4)	12.3	–	–	
**47**	–	652 (10)	9.9	–	–	
**50**	571 (37)	602 (12)	72	8.8/2.6× 10^5	–	[48–50]

Table S2. Spectral-fluorescent properties of trimethine cyanine dyes in a complex with BSA (in the presence of high concentrations of BSA): maxima of the absorption and fluorescence spectra λ_{abs}(BSA) and λ_{fl}(BSA), respectively, spectral shifts upon the interaction with BSA $\Delta\lambda_{abs}$ and $\Delta\lambda_{fl}$, ratios of fluorescence intensities /quantum yields in the presence and in the absence of BSA I_{fl}(BSA)/I_{fl0} or Φ_{fl}(BSA)/Φ_{fl0}; as well as dye–BSA binding constants K_b and limits of detection/quantification of BSA LOD/LOQ using the dyes as probes

Dye	λ_{abs}(BSA) (~$\Delta\lambda_{abs}$)	λ_{fl}(BSA) (~$\Delta\lambda_{fl}$)	I_{fl}(BSA)/I_{fl0} Φ_{fl}(BSA)/Φ_{fl0}	K_b	LOD/LOQ	Ref
	nm	nm		L mol^{-1}	μg mL^{-1}	
**1**	535 (0)	572 (8)	0.22	–	–	[27]
**2**	551 (-3)	572 (-5)	0.002	–	–	
**4**	562 (9)	661 (4)	3.57	–	–	
**5**	577 (29)	668 (25)	21.5	–	–	
**6**	501 (–)	638 (2)	3.75	–	–	
**7**	525 (-2)	578 (–)	1.1	–	–	
**8**	557 (-1)	601 (-114)	2.09	–	–	
**9**	524 (28)	596 (-33)	3.47	–	–	
**10**	509 (9)	569 (-86)	11	–	–	
**11**	510 (7)	655 (-17)	5.25	–	–	
**12**	528 (1)	655 (33)	10.6	–	–	
**13**	518 (-10)	632 (3)	0.678	–	–	
**46**	618 (46)	653 (11)	7.90	–	–	[65]
**47**	607 (1)	638 (10)	1.20	–	–	
**52**	584	608	100	3.5×10^5	5.4/16.6	[79]
**58**	670 (12)	695 (22)	19.5	–	–	[65]
**59**	557 (7)	590 (15)	14.1	–	–	
**69**	509 (7)	525 (10)	16	$1.45 – 1.59 \times 10^5$	–	[75]
**70**	680 (14)	701 (1)	11	$1.83 – 3.02 \times 10^5$	–	[77]
**71**	678 (14)	699 (-1)	7.75	$7.6 – 9.2 \times 10^4$	–	
**72**	674 (12)	700 (2)	10.3	$3.14 – 3.88 \times 10^4$	–	
**73**	675 (13)	702 (6)	7.75	$1.26 – 1.71 \times 10^4$	–	
**79**	596 (20)	596 (-13)	53	4.1×10^5	1.73/5.7	[79]
**80**	570 (10)	597 (14)	41	4.9×10^4	5.8/17.3	

Table S3. Spectral-fluorescent properties of trimethine cyanine dyes in a complex with HSA (in the presence of high concentrations of HSA): maxima of the absorption and fluorescence spectra λ_{abs}(HSA) and λ_{fl}(HSA), respectively, spectral shifts upon the interaction with HSA $\Delta\lambda_{abs}$ and $\Delta\lambda_{fl}$, ratios of fluorescence intensities/quantum yields in the presence and in the absence of HSA I_{fl}(HSA)/I_{fl0} or Φ_{fl}(BSA)/Φ_{fl0}; as well as dye–HSA binding constants K_b and limits of detection/quantification of HSA LOD/LOQ using the dyes as probes

**Dye**	**λ_{abs}(HSA) (~$\Delta\lambda_{abs}$)**	**λ_{fl}(HSA) (~$\Delta\lambda_{fl}$)**	**I_{fl}(HSA)/I_{fl0} Φ_{fl}(HSA)/Φ_{fl0}**	**K_b**	**LOD/LOQ**	**Ref.**
	nm			L mol^{-1}	nmol L^{-1}	
**15**	565 (12)	–	–	1.05 – 1.74 × 10^4	–	[15]
**19**	563 (21)	–	–	1.0 × 10^4	–	
**52**	604 (12)	616 (-44)	885	3.7 × 10^7	0.79/2.6	[78, 79]
**53**	610 (11)	–	718	>10^6	1.83/6.0	[15, 78]
**60**	558 (10)	–	–	3.0 – 6.0 × 10^7	–	[15]
**61**	574 (12)	–	–	4.9 – 5 × 10^7	–	
**64**	567 (10)	–	–	3.5 × 10^3	–	
**66**	566 (9)	–	–	2.0 – 3.0 × 10^6	–	
**69**	513 (15)	529 (15)	15.5	2.23 × 10^5	32/105	[74, 78]
**78**	563 (11)	–	45.4	1.09 × 10^5	4.2/14.7	[78]
**79**	606 (30)	620 (11)	119	4.3 × 10^5	1.4/4.6*	[79]
**80**	592 (32)	606 (23)	183	2.1 × 10^6	0.35/1.14*	
**82**	529 (-27)	561	–	7.54 × 10^5	–	[84]
**83**	531 (-25)	605	–	8.75 × 10^5	–	
**84**	532 (-31)	565	–	8.03 × 10^5	–	
**85**	569 (-27)	603	–	3.25 × 10^5	–	
**86**	572 (-28)	605	–	8.33 × 10^5	–	
**87**	572 (-45)	607	–	4.13 × 10^5	–	
**88**	764 (-4)	771	–	1.55 × 10^5	–	
**89**	758 (-12)	774	–	1.06 × 10^6	–	
**90**	758 (-15)	775	–	3.51 × 10^5	–	

* In μg mL^{-1}
